# Dynein disruption perturbs post-synaptic components and contributes to impaired MuSK clustering at the NMJ: implication in ALS

**DOI:** 10.1038/srep27804

**Published:** 2016-06-10

**Authors:** Valérie Vilmont, Bruno Cadot, Elsa Vezin, Fabien Le Grand, Edgar R. Gomes

**Affiliations:** 1 Myology Research Center, UM76-INSERM U974-CNRS FRE 3617 Sorbonne Universités, UPMC Université Paris 06, Paris, France; 2 Instituto de Medicina Molecular, Faculdade de Medicina, Universidade de Lisboa, Lisboa, Portugal

## Abstract

The neuromuscular junction (NMJ) allows the transformation of a neuronal message into a mechanical force by muscle contraction and is the target of several neuromuscular disorders. While the neuronal side is under extensive research, the muscle appeared recently to have a growing role in the formation and integrity of the neuromuscular junction. We used an *in vitro* model of mature myofibers to study the role of dynein on major postsynaptic proteins. We found that dynein affects the expression and the clustering of acetylcholine receptors (AChRs), muscle specific tyrosine kinase (MuSK) and Rapsyn. We also show that myofibers with dynein impairment or from an amyotrophic lateral sclerosis (ALS) model (SOD1^G93A^) show similar defects in myofiber formation and agrin-induced AChR clustering suggesting a role for dynein impairment in ALS progression. Finally, we found that dynein can affect MuSK traffic through the endosomal pathway. Collectively, our studies show that defects in dynein can lead to impairment of muscle NMJ components’ expression and clustering. We propose that NMJ defects could happen via defective MuSK traffic and that this could be one of the pathological features involved in neurodegeneration such as ALS.

The NMJ is a structure at the basis of synapse-dependent muscle contraction where the motor neuron interacts with the muscle^1^,^2^. At the molecular level of the vertebrate NMJ, the muscle specific tyrosine kinase (MuSK) and its co-receptor Lrp4^3^,^4^,^5^,^6^,^7^,^8^, at the post-synapse, are the key orchestrators of the NMJ formation and maintenance. Neuronal agrin, an heparan sulfate proteoglycan, once secreted, will bind to Lrp4 and potentiate the binding to MuSK and MuSK kinase activity^9^.

Transport along the axon is important for synapse formation and dynein, a microtubule motor is involved in such transport and the maintenance of synapses^10^,^11^. Dynein is also important for golgi integrity^12^ and endosomal recycling pathway^12^. Dynein dysfunction leads to defects of neuromuscular synapses^13^ which can result in motor neuron degeneration^14^,^15^,^16^,^17^, and ALS^17^,^18^.

While much attention has been given to the motor neuron in ALS^19^,^20^,^21^, muscle impairment can also be important for ALS^22^,^23^,^24^,^25^. Indeed, one of the earliest signs of ALS pathobiology is altered muscle metabolism^24^,^26^,^27^. This takes place before any motor neuron degeneration. Furthermore, over-expression of MuSK in muscle delayed denervation and improved motor function in ALS mice^28^.

Because the dynein complex has been described as an important protagonist of muscle development^29^,^30^,^31^, we investigated if muscle dynein is involved in NMJ formation and in ALS. To address this issue, we used highly differentiated *in-vitro* myofibers^32^. Through the use of shRNA and drugs, we specifically impaired dynein during differentiation of myofibers. We found that the overall muscle differentiation process and differentiation of the post-synapse and the maintenance of NMJs are dependent on dynein. The latter is involved in the correct localization of MuSK during endosomal trafficking. Similarly, impaired localization of MuSK was also observed in ALS muscle fibers. Therefore we conclude that the NMJ loss in ALS or in dynein-related neuromuscular disorders can be due in part to a defect in MuSK turnover at the NMJ.

## Results and Discussion

### Dynein is involved in AChR cluster formation and maintenance

**Figure 1:**
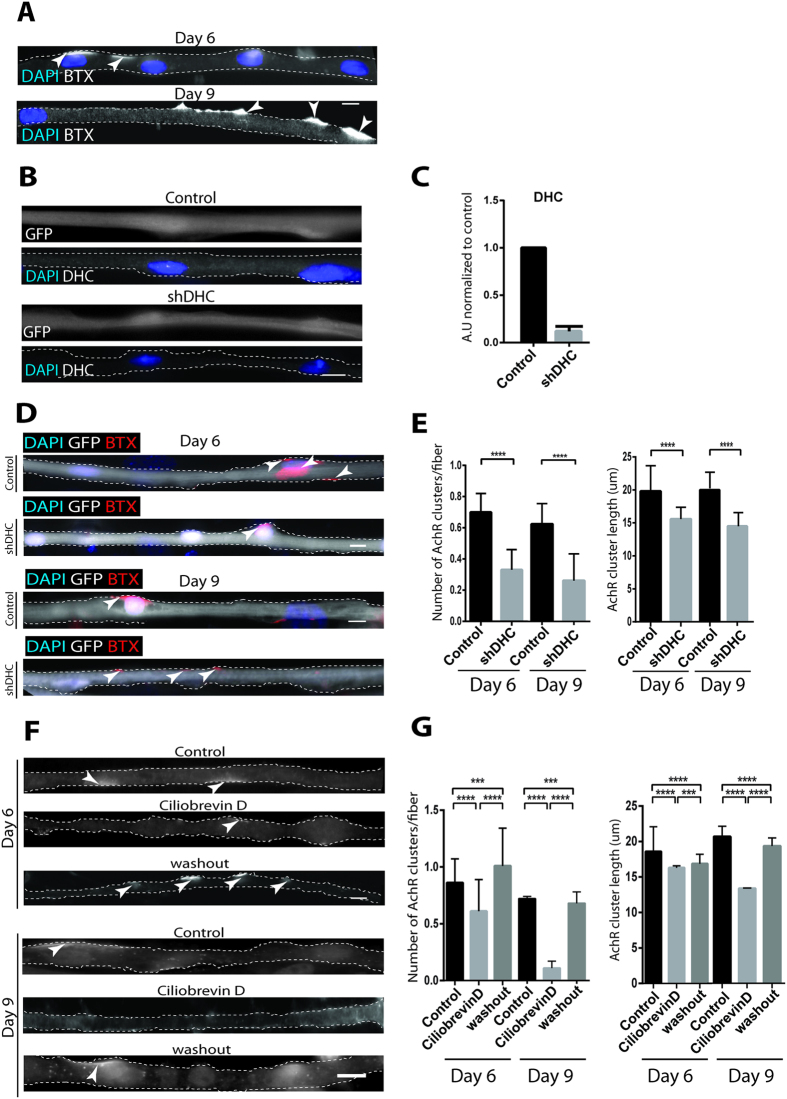
Dynein is involved in AChR clusters formation and maintenance. (**A**) Representative epifluorescence images of AChR clusters stained with TRITC, α-Bungarotoxin (BTX) and nuclei stained with DAPI (nucleus) at day 6 and day 9 of myofiber differentiation. Myofiber boundaries determined from brightfield images (not shown) are indicated by dashed lines. (**B**) Representative epifluorescence images myofibers at day 9 of differentiation transfected with control GFP plasmid (Control, top) or DHC *sh*RNA plasmid (*sh*DHC, bottom), both expressing GFP. Myofibers where stained for GFP or DHC, as indicated. Myofiber boundaries are indicated by dashed lines. Note that DHC intensity is decreased in *sh*DHC condition. (**C**) RT-qPCR analysis of gene expression in *in-vitro* differentiated myofibers after *sh*RNA treatment. Dynein heavy chain expression show efficient reduction of the transcription of genes targeted by *sh*DHC compared to scramble transfected cells. Error bars indicate SEM. (**D**) Representative epifluorescence images of myofibers at day 6 or day 9 of differentiation transfected with control GFP (Control) or DHC *sh*RNA plasmid (*sh*DHC) and stained for AChR with BTX (red) GFP (gray) and DAPI (blue). Myofiber boundaries are indicated by dashed lines. (**E**) Quantification of the number of AChR clusters per fiber and AChR cluster length in control or *sh*DHC conditions, at two differentiation time points (days 6 and 9). (**F**) Representative epifluoresence images of AChR clusters in control, ciliobrevinD-treated (Day 1 or 4) and myofibers washout from ciliobrevinD (Day 4 or Day 7). Day 6 panel: fibers in all conditions were fixed and stained at day 6. Day 9 panel: fibers in all conditions were fixed and stained at day 9 of differentiation. BTX (Gray) and DAPI (blue). Myofiber boundaries are indicated by dashed lines. (**G**) Quantification of AChR clusters number per fiber and AChR cluster length in ciliobrevinD washout assay at 2 differentiation time points (days 6 and 9). Scale bar in (**A–C,E**), 10 μm.

We differentiated myofibers from primary myoblasts isolated from WT or histone2B-GFP (H2B-GFP) P7 mice as previously described^32^. We used neural agrin known to induce acetylcholine receptor (AChR) clustering, a post-synaptic receptor expressed at NMJs *in vivo*^33^,^34^,^35^,^36^. AChR clusters were defined as bright large patches of positive rhodamine labeled α-bungarotoxin at day 6 and 9 of differentiation along the myofibers axis (Fig. 1A)^33^,^37^,^38^. We also verified the presence and clustering of Rapsyn, another post-synaptic protein, that colocalized with AChR clusters (Supplementary Fig. 1A)^39^,^40^,^41^,^42^.

To determine the role of dynein on the formation of AChR clusters, we transfected myoblasts with an *shRNA* directed against dynein heavy chain (DHC) that efficiently decreased the level of DHC in day 9 myofibers (Fig. 1B,C)^43^. A decrease of the levels of intermediate chain (DIC) upon DHC shRNA transfection was also observed as well as Golgi dispersal in mononucleated and in undifferentiated muscle cells, as previously described (Supplementary Fig. 1B–D)^43^,^44^. At days 6 and 9 of differentiation, we found that the number and the length of AChR and Rapsyn clusters per fiber were significantly reduced in agrin-treated *sh*DHC-transfected myofibers compared to control (Fig. 1D,E, Supplementary Fig. 1E). Interestingly, we found that *sh*DHC caused a significant decrease in AChR and Rapsyn expression at mRNA levels, in line with a decrease in number and length of clusters (Supplementary Fig. 1F,G). Dynein is a motor protein that uses ATP as energy source to generate movement^45^. To determine the involvement of dynein ATPase and motor function in AChR cluster formation we used the dynein specific ATPase inhibitor ciliobrevinD^46^. CiliobrevinD was added at Day 1 or Day 4 of differentiation and left for 72 hours before ciliobrevinD washout from cells and fixation at Day 6 or Day 9, respectively (Supplementary Fig. 1H). At days 6 and 9 of differentiation, ciliobrevinD induced a significant decrease in AChR and Rapsyn cluster number and length in myofibers compared to DMSO-treated myofibers (Fig. 1F,G, Supplementary Fig. 1I). Upon ciliobrevinD washout, numbers and length of AChR or Rapsyn clusters were significantly rescued (Fig.1F,G and Supplementary Fig. 1I). CiliobrevinD exposure did not have any effect on dynein expression or on microtubules longitudinal organization (Supplementary Fig. 2A,B). To rule out the possibility that effect on AChR and Rapsyn clustering upon *sh*DHC or ciliobrevinD was not due to alteration in general muscle differentiation features, we checked markers of muscle differentiation in *sh*DHC-transfected and ciliobrevinD treated cells at Day 6. We immunostained our cells for muscle myosin heavy chain, a marker of differentiation, and α-actinin, which is found at the z-line, to assess the correct assembly of sarcomeres. We found that α-actinin expression and striated localization was unchanged in *sh*DHC or unreleased conditions compared to control conditions (Supplementary Fig. 2C,D). Similarly no difference was seen in localization and expression of myosin heavy chain in shDHC or ciliobrevinD washout assay conditions (Supplementary Fig. 2E,F). However, we cannot rule out the possibility that at late time-points of differentiation (day 9), the impairment in AChR and Rapsyn clusters formation that we observed could be either i) a consequence of possible general muscle differentiation defaults induced by dynein disruption or ii) enhanced by the delayed muscle differentiation. Thus, our results suggest a role for dynein in the formation of agrin-induced AChR and Rapsyn clusters during myofiber differentiation.

### Dynein is important for MuSK localization and clustering at AChR clusters

Agrin is known to stimulate *in vivo* the activity of MuSK via Lrp4^47^. In turn, MuSK, triggers various intracellular pathways among which stabilization of AChR clusters, forming therefore a positive feedback loop allowing post-synaptic and presynaptic differentiation^48^. In absence of MuSK, muscle fibers do not form AChR clusters or NMJs^3^,^5^,^49^.

**Figure 2:**
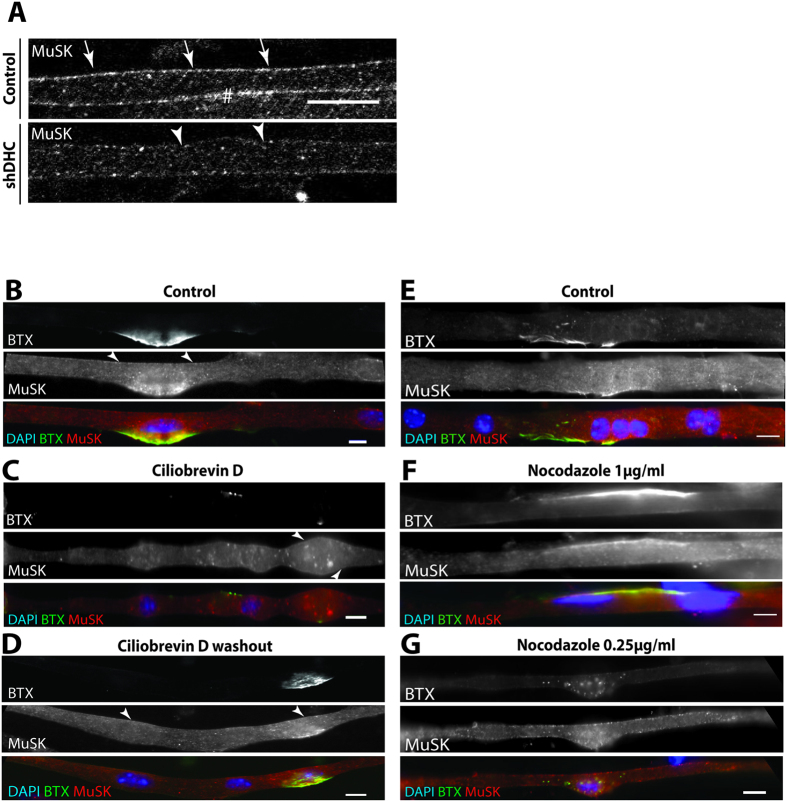
Dynein is involved in MuSK localization at cell periphery and clustering at NMJ and microtubules are important for stabilization of NMJ. (**A**) Representative confocal images of day 9 myofibers transfected with control GFP plasmid (Control, top) or DHC *sh*RNA (*sh*DHC, bottom) and stained for MuSK. Arrows show MuSK at cell periphery. Note that MuSK localization at the cell periphery is reduced in *sh*DHC-transfected myofiber. (**B**–**D**) Representative epifluorescence images of myofibers: not treated with ciliobrevinD ((**B**); control), treated with ciliobrevinD from Day 1 to Day 6 of differentiation (**C**, ciliobrevinD) or after ciliobrevinD washout at Day 4 of differentiation ((**D**), washout) as indicated. All conditions were fixed and stained at Day 6 of differentiation. Myofibers are stained for AChR with BTX (green), MuSK (red) and DAPI (blue). Arrowheads show MuSK localization at cell periphery. (**E**) Representative epifluorescence images of myofibers in control conditions stained for AChR with BTX (green), MuSK (red) and DAPI (blue).(**F**) Representative epifluorescence images of myofibers treated with nocodazole at Day 4 of differentiation for 2 hours (1 μg/ml) stained for α-BTX (green), MuSK (Red) and DAPI (blue). Arrows show MuSK localization at cell periphery. (**G**) Representative epifluorescence images of myofibers treated with nocodazole from Day 4 to 7 of differentiation (0.25 μg/ml) stained for α-BTX (green), MuSK (Red) and DAPI (blue). Scale bar (**A**) 20 μm. Scale bar (**B–G**) 10 μm.

We investigated the role of dynein on MuSK recruitment to the plasma membrane of myofibers. Downregulation of dynein through shRNA reduced MuSK localization at the plasma membrane compared to a scramble shRNA on Day 9 myofibers (Fig. 2A). qPCR results revealed that expression of MuSK was decreased in *sh*DHC conditions compared to control (Supplementary Fig. 3A). Decrease of MuSK expression could explain the decrease of the tyrosine kinase at the membrane. Further experiments would be needed to shed light on how downregulation of the expression a motor protein like dynein could lead to a decrease in MuSK mRNA. It is possible that dynein downregulation affects, in the first place, general metabolism pathways like mitochondrial physiology^50^ or general muscle differentiation pathways^29^ that would in turn affect the expression of MuSK. In fact it is known that regulation of MuSK is dependent on muscle differentiation, but to which extent could dynein be playing a role in this crosstalk is presently unknown.

On the other hand, ciliobrevinD exposure from Day 1 to Day 6 did not affect MuSK localization at plasma membrane. However, it inhibited its localization at AChR clusters (Fig. 2B–D)^51^,^52^ and induced formation of cytoplasmic aggregates, which disappear upon ciliobrevinD washout. After washout, MuSK colocalized again with AChR clusters (Fig. 2D). We noticed that, although nearly all the myofibers recovered a normal structure, treatment with ciliobrevinD at such concentration affected to some extent the structure of some myofibers. Therefore we asked whether the formation of MuSK aggregates was a consequence of altered structure or dynein motor-dysfunction. We found that in myofibers, washed out from ciliobrevinD, which still show some structure alteration, did not form any MuSK aggregates and could cluster, supporting the notion that aggregation was not a consequence of cell structure distortion (Supplementary Fig. 3B). However, we cannot exclude the possibility that structural changes in the presence of ciliobrevinD are not a consequence of general structural changes in myofibers. The differences between *sh*DHC and ciliobrevinD treatments could be due to their intrinsic properties: *sh*DHC effect is more latent given its dependence on the cell endogenous machinery whereas ciliobrevinD is highly permeant and acts independently of any cell machinery. Additionally, we cannot rule out that the differences in effect of shDHC vs ciliobrevin might also be due in part to possible ciliobrevinD off-targets^46^,^53^. Surface MuSK which is highly stabilized naturally could be affected only in conditions of potent dynein downregulation which happens with *sh*DHC. However, MuSK clusters which are only found at NMJs, are known to be labile, MuSK being internalized after its trans-phoshorylation^6^ could be potentially disassembled in circumstances of sole dynein motor defect without any downregulation of dynein expression. Nevertheless, our results described a decisive role for dynein in MuSK localization in myofibers.

Given that dynein is a microtubule motor, we next sought to examine whether microtubules were necessary for the colocalization of MuSK with AChR clusters. To this end, we used two different conditions of nocodazole treatment: a high dose of nocodazole (1 μg/ml) for a short period of time (2 h) or a lower dose of nocodazole (0.25 μg/ml) for a long period (3 days). As expected, MuSK colocalized with AChR clusters in DMSO-treated myofibers (Fig. 2E). At high doses of nocodazole, the microtubule network is completely depolymerized with no stable microtubules left (Supplementary Fig. 3C,D). In such conditions, MuSK was still found at AChR clusters in myofibers suggesting that over short time periods, microtubules might not be important for the maintenance of the clusters, probably because the half-life of these clusters is longer than two hours^37^ (Fig. 2F). However, because we did not assess the number of clusters before and after treatment with nocodazole, we cannot exclude the possibility that disrupted microtubules network might have impaired the formation of new clusters. At low doses, microtubules network was also completely affected and allowed us to interfere with MTs for a longer period of time than higher doses (Supplementary Fig. 3E). In these conditions, both MuSK clusters and AChR clusters disappeared from the plasma membrane and were found as small aggregates in the cytoplasm (Fig. 2G). After a recovery period of two days after nocodazole washout from low dose treatment, MuSK and AChR clusters were still not formed (Supplementary Fig. 3F). These results suggest that over longer periods of time in the absence of microtubules (more than 2 days), clusters disappear and cannot reform. It is also plausible that clusters could reform but could not be maintained and therefore could not be visualized. Possible explanations could be: i) microtubule network might not have been being totally built and stabilized or ii) severe microtubule disruption might have altered other structures that could not properly re-formed even upon microtubule regrowth. Altogether these results suggest that microtubules are probably important for the maintenance of MuSK clusters at the plasma membrane over long time periods but not over short periods.

Dynein mutant mice show neurodegeneration alongside loss of α-motor neurons and alteration of fiber morphology indicating the important role of dynein at the pre- and post-synapse^15^. Likewise, destabilization of microtubule network has been directly linked to ALS^52^,^53^. Altogether, our results posits a potential explanation to those phenotypes through the loss of the master organizer of the NMJ, MuSK, following dynein altered motor activity or loss of microtubule integrity.

### AChR clusters and MuSK are affected in neurodegenerative conditions where dynein function is known to be impaired

Transport along the axonal microtubules mediated by dynein and kinesins is important for synapse formation^54^. Dynein has been associated to neuronal defects in Drosophila^55^, to neurodegeneration in mice^16^, to a slowly progressive motor neuron disease together with muscle atrophy and weakness in both human and mice^17^ and in amyotrophic lateral sclerosis (ALS)^18^. In all these instances, many biological processes dependent on dynein motor are impaired such as retrograde axonal transport^56^,^57^, Golgi integrity^12^ or endosomal recycling pathway^12^. These studies supported the idea that the motor neuron is the most important protagonists of ALS pathogenesis. However, other recent works suggest that the muscle could trigger, prior to the neuron, the disease and have shown that supplementing important post-synaptic proteins to ALS mice can ameliorate motor function^23^,^25^,^28^.

Based on our results, we reasoned that in neurodegenerative conditions such as ALS, where dynein complex is known to be impaired, loss of NMJ integrity could be due to the role of dynein in the muscle by regulating AChR cluster dynamics and MuSK localization^18^,^55^,^56^,^58^,^59^. To test this idea, we investigated AChRs clusters and MuSK expression in our *in-vitro* model using SOD1^G93A^ P7 asymptomatic-mice myoblasts and in isolated fibers from the extensor digitorum longus (EDL) of symptomatic SOD1^G93A^ mice. This mouse model has been widely used to study ALS^55^,^56^,^57^,^58^,^59^, where misfolded SOD1 protein has been shown to aggregate the dynein complex and hence block its normal motor function^57^,^60^.

**Figure 3:**
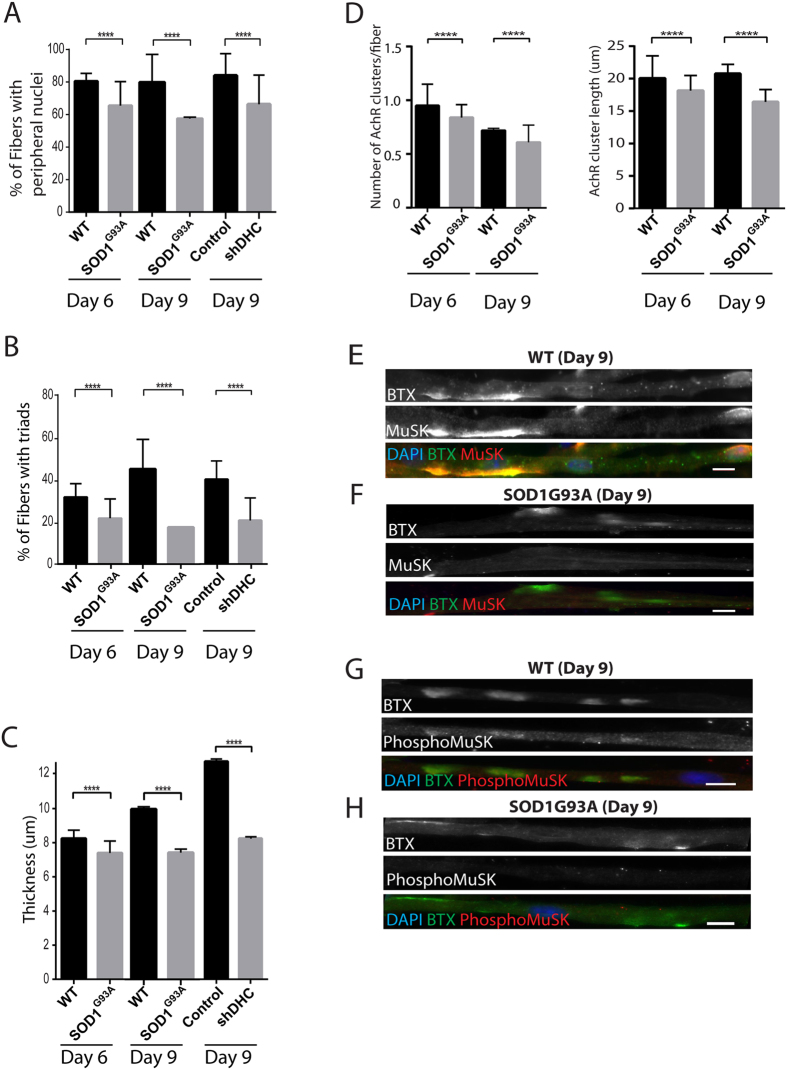
SOD1^G93A^* in-vitro* differentiated myofibers show similar defects to shDHC myofibers. (**A**) Quantification of peripheral nuclei in WT and SOD1^G93A^ myofibersat days 6 and 9 of differentiation, in control and shDHC at day 9 of differentiation. (**B**) Quantification of myofibers with triads in WT and SOD1^G93A^ conditions at days 6 and 9 of differentiation, and in control and shDHC at day 9 of differentiation. (**C**) Quantification of myofiber thickness in WT and SOD1^G93A^ conditions at days 6 and 9 of differentiation, and in control and shDHC transfected myofibers at day 9 of differentiation. (**D**) Quantification of number of AChR cluster per fiber and AChR cluster length in WT and SOD1^G93A^ conditions at days 6 and 9 of differentiation. (**E**) Representative epifluorescence images of WT myofibers at day 9 stained for AChR with BTX (green), MuSK (Red) and DAPI (blue). (**F**) Representative images of SOD1^G93A^ myofibers at day 9 stained for α-BTX (green), MuSK (Red) and DAPI (blue). (**G**) Representative epifluorescence images of WT myofibers at day 9 stained for α-BTX (green), phosphoMuSK (Red) and DAPI (blue). (**H**) Representative epifluorescence images of SOD1^G93A^ myofibers at day 9 stained for α-BTX (green), phosphoMuSK (Red) and DAPI (blue). Scale bar 10 μm.

We investigated myofiber maturation through the measurement of three parameters: i) peripheral nuclei; ii) transversal triads, iii) myofiber thickness^32^. We observed a significant reduction in peripheral nuclei, triad formation and thickness in SOD1^G93A^ compared to SOD1^wt^ at days 6 and 9 of differentiation (Fig. 3A–C)^61^. These results are consistent with conditions where dynein expression is down-regulated by *sh*DHC compared to control cells (Fig. 3A–C). Number and length of AChR clusters were also decreased in SOD1^G93A^ when compared to SOD1^wt^ after 6 days of differentiation (Fig. 3D) and 9 days of differentiation (Fig. 3D).

To further assess NMJ formation defects, we analyzed MuSK, active MuSK and Rapsyn distribution. It has been reported that following agrin-Lrp4-MuSK signaling at NMJ, MuSK is activated by phosphorylation that is important for downstream signaling, differentiation and stabilization of the NMJ^62^. We found that after 6 days in differentiation, MuSK, active MuSK (phosphoMuSK) and Rapsyn were at AChR-rich clusters in both the SOD1^wt^ and SOD1^G93A^ (Supplementary Fig. 4A–C). However, after 9 days of differentiation, MuSK and active MuSK were not present in AChR-rich clusters in SOD1^G93A^ compared to SOD1^WT^ (Fig. 3E–H). Thus these results show that myofibers with dynein impairment or from an ALS model (SOD1^G93A^) show similar defects on myofiber formation and agrin-induced AChR clusters. Interestingly, because these SOD1^G93A^ show same myofiber differentiation defects as in *sh*DHC at late differentiation (Day 9), it could be possible that the defective phenotypes observed for post-synaptic features like clusters of MuSK and active MuSK are either i) subsequent to an underlying myofiber differentiation impairment or ii) already triggered by dynein dysfunction at earlier time points (Day 6) and enhanced in these mice due to underlying muscle differentiation defects linked to dynein dysfunction. Another interesting possibility would be that defective MuSK clustering could itself impair muscle differentiation. Collectively, these results suggest that dynein impairment can be one of the many causes of the defects observed at the NMJ in the neurodegenerative conditions in ALS.

**Figure 4:**
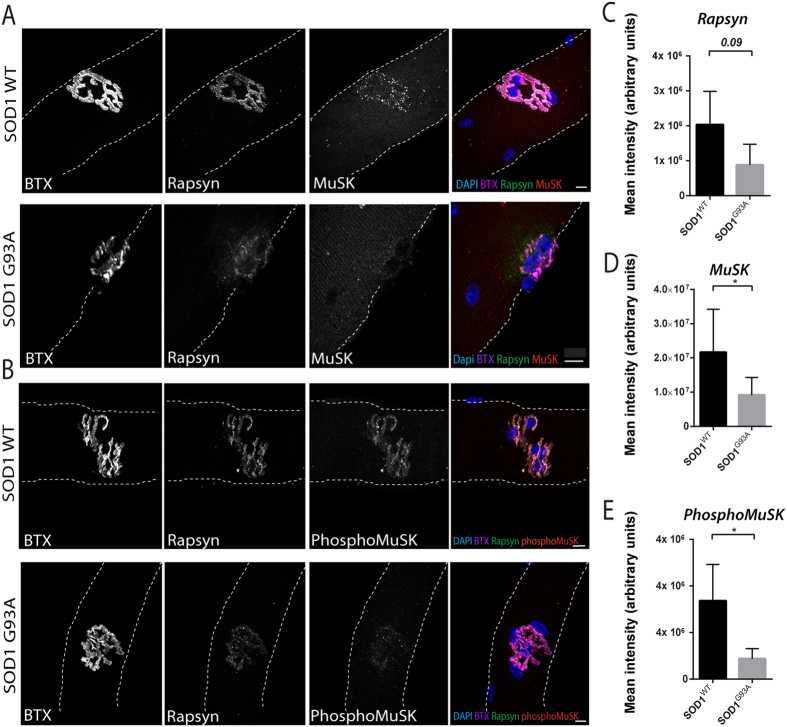
Loss of MuSK, phosphoMuSK and Rapsyn in SOD1^G93A^ fibers *in vivo*. (**A**) Representative confocal images of fibers in SOD1^WT^ and SOD1^G93A^ isolated fibers stained for α-BTX (magenta), rapsyn (green), MuSK (red) and DAPI (blue). Myofiber boundaries are represented as dash lines. (**B**) Representative confocal images of fibers in SOD1^WT^ and SOD1^G93A^ isolated fibers stained for α-BTX (magenta), rapsyn (green), phosphoMuSK (red) and DAPI (blue). Myofiber boundaries are represented as dash lines. (**C**) Mean intensity of MuSK signal in SOD1^WT^ vs SOD1^G93A^ isolated fiber. (**D**) Mean intensity of Rapsyn signal in SOD1^WT^ vs SOD1^G93A^ isolated fiber. (**E**) Mean intensity of phosphoMuSK signal in SOD1^WT^ vs SOD1^G93A^ isolated fiber. Scale bar 10 μm.

Next, we looked at motor endplates of EDL muscles from 120 day mice. In SOD1^wt^ isolated fibers, the motor endplates appeared as round pretzel-like arrangements of tubular junctional folds (Fig. 4A), positive for Rapsyn, MuSK and active MuSK (Fig. 4A,B). In SOD1^G93A^ isolated fibers, we found frequently condensed and fragmented pretzel-like AChRs structures (Fig. 4A,B)^63^ with a significantly lower amount of MuSK and active MuSK (Fig. 4C–E). The reduction of active MuSK could be explained by the observed loss of MuSK itself and suggested that active MuSK was not necessarily fixed at the motor-end plate but rather dynamic and dependent on the pool of MuSK. This explanation is supported by evidence that once MuSK is phosphorylated it will be endocytosed to be further recycled^48^.

Our findings revealed that muscle development and features of the post-synaptic apparatus are impaired in SOD1^G93A^ fibers taken from young mice which are still asymptomatic and grown aneurally. In such conditions muscle fibers seem to be active protagonists in ALS progression taking into account the absence of any neuronal signal. However, regarding the phenotypes of the post-synaptic structures and muscle atrophy witnessed in *ex vivo* SOD1^G93A^ mice fibers, we cannot exclude a role of denervation given that at 120 days, axon have already started retracting. While MuSK and Rapsyn are known to induce and stabilize AChR clusters, our results suggest that their failure to cluster properly might be the primary features of NMJ destabilization. These proteins are present at the post-synapse before the arrival of the neurons^9^. It is therefore highly possible that their absence induces negative retrograde signals to the neurons. It supports the hypothesis that muscle development can be one of the triggers of neuromuscular junction loss and muscle atrophy^23^,^28^.

### MuSK loss at the NMJ is linked to impaired trafficking via dynein-dependent endosomal trafficking

**Figure 5:**
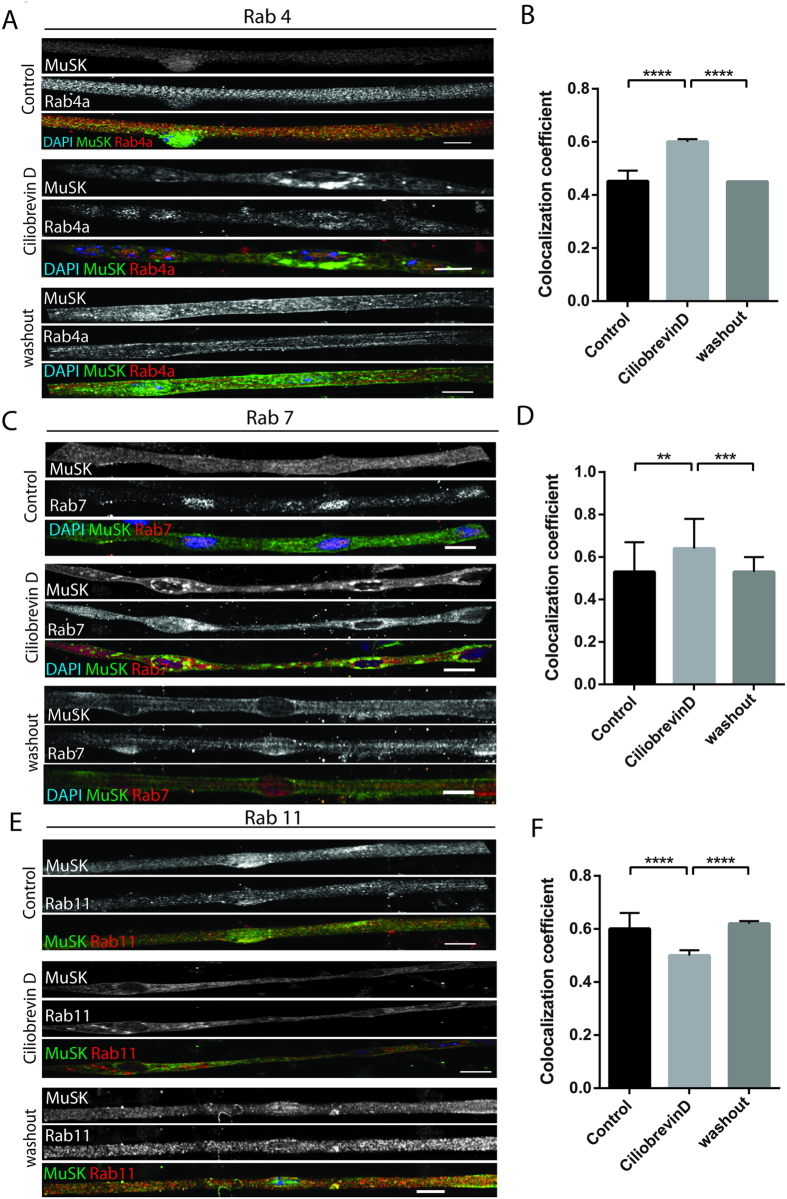
Stabilization of MuSK clusters at NMJ depends on Rab-mediated dynein dependent trafficking. (**A**) Representative epifluorescence images of myofibers in control, ciliobrevinD-treated and washout conditions stained for MuSK (green), Rab4a (Red) and DAPI (blue). (**B**) Quantification of colocalization coefficients between MuSK and Rab4a signals ciliobrevinD washout assay. (**C**) Representative epifluorescence images of myofibers in control, ciliobrevinD-treated and washout conditions stained for MuSK (green), Rab7 (Red) and DAPI (blue). (**D**) Quantification of colocalization coefficients between MuSK and Rab4a signals ciliobrevinD washout assay.(**E**) Representative epifluorescence images of myofibers in control, ciliobrevinD-treated and washout conditions stained for MuSK (green), Rab11 (Red) and DAPI (blue). (**F**) Quantification of colocalization coefficients between MuSK and Rab11 signals ciliobrevinD washout assay. Scale bar 10 μm.

MuSK endocytosis and recycling via Rab11-endosomes is important for the initiation of synapse formation^64^,^65^. Dynein complex is involved in endosome trafficking^56^,^66^,^67^,^68^,^69^. We hypothesized that dynein could be important in MuSK endocytosis and recycling via the endosomal pathway. Accordingly, we conducted ciliobrevinD experiments as described in Fig. 2 and examined the distribution of MuSK within the early, late and exocytic/recycling endosome compartments, labeled by Rab4, Rab7 and Rab11 antibodies respectively. The different Rab stainings showed a punctate phenotype distributed throughout the entire myofibers (Fig. 5A,C,E). As expected, the distribution of the different endosomal compartments was altered in the presence of ciliobrevinD^70^.

Focusing on regions of MuSK accumulation, we found a higher colocalization of MuSK with early endosomes (Rab4) and late endosomes (Rab7) in ciliobrevinD conditions compared to control, at day 6 (Fig. 5A–D). After ciliobrevinD washout, MuSK showed a decrease in early and late endosome colocalization, similar to a control situation (Fig. 5A–D). Colocalization of exocytic/recycling-endosome (Rab11) and MuSK was decreased upon treatment with ciliobrevinD when compared to control (Fig. 5E,F). Colocalization returned to control levels in myofibers washed out from ciliobrevinD at Day 4 (Fig. 5E,F).

Taken together, these results suggest that in conditions of dynein motor dysfunction, MuSK distribution in endosomal compartment which has been correlated to MuSK recycling status^65^ is unbalanced. In conditions where dynein activity is impaired, MuSK accumulates in early and late endosomes and is decreased in recycling endosomes. Therefore the role of dynein in MuSK transport is probably important for NMJ formation and maintenance since MuSK is continuously recycled to the surface at future synaptic sites^64^. Moreover, given the importance of MuSK in AChR clustering^2^, the decrease which we observed in AChR clusters in ciliobrevinD conditions (Fig. 1G) could be a consequence of a disequilibrium in MuSK recycling.

Overall, our data suggests a role of dynein in MuSK recycling and transport which can probably lead consequently to gradual Rapsyn destabilization from the NMJ and further AChR cluster loss like seen in SOD1^G93A^ mice. Such events occurring at the post-synapse replace the muscle as a primary target for ALS treatment. Whether MuSK defective trafficking can be enhanced due to other impaired processes such as delayed differentiation disrupted in conditions of dynein dysfunction still needs to be investigated and the role of denervation *in vivo* needs further studies.

## Materials and Methods

### Animals

All experimental protocols and tissue collected were approved by the animal facility of Pierre et Marie Curie University and carried out in accordance with the Animal Ethics Committee guidelines. SOD1G93A (originally named B6SJL-Tg (SOD1-G93A)1Gur/J) mice were obtained from the Jackson Laboratory. Histone 2B-GFP (H2B6GFP) have been previously described^32^. Primary murine myoblasts were obtained from animals of either sex.

### Reagents

Matrigel Basement Membrane Matrix was purchased at Corning Life Sciences (ref 354230). Matrigel protein concentration as obtained by Lowry method ranged between 9.2 and 10.4 mg/ml and endotoxin as measured by Limulus Amoebocyte lysate assay was less than 1.5 EU/ml. Collagenase, TRITC-α-Bungarotoxin were purchased from Sigma (ref C9263, M1404 and ref T0195 respectively). Dispase II was purchased from Roche (Neutral protease, grade II, ref 04942078001). Chicken Embryo Extract was purchased at MP Biochemicals (MP Biochemicals ref 2850145). CiliobrevinD was purchased at Merck Millipore (ref 250401). Recombinant rat agrin was purchased at R&D Systems (ref 550-AG-100).

### Primary skeletal muscle culture and differentiation

Skeletal muscle cultures from H2B-GFP mice has been previously described^32^. Skeletal muscle cultures form SOD1G93A mice were performed as previously described^32^ and with the following alterations: Tibialis anterior, extensor digitorum longus, gastrocnemius and quadriceps from thigh muscles were sampled from P7 pups. Pre-plating was done over 3 hours. IBIDI plates suitable for immunofluorescence (Biovalley, ibi-Treat μplate, 96-well, ref 89626) were coated with Matrigel to 1/3 dilution. Once primary myoblasts have reached 60–70% confluence, cells were switched to differentiation medium consisting of IMDM with Glutamax 1% penicillin/streptomycin 2% Horse serum supplemented with agrin (100 ng/ml). The day after, myotubes are coated with Matrigel and kept in fresh differentiation medium.

### shRNAs and transfection

shDHC was a gift from Richard B. Vallee and has been previously described^43^. Once primary myoblasts have reached 60–70% confluence, cells were transfected with Lipofectamine 2000 (Life Technologies, ref 11668–019) with either *sh*DHC or *sh*GFP. Cells were incubated with liposome-shRNA complex for 6 hours before being washed twice and switched to differentiation medium. The day after, myotubes were coated with Matrigel and kept in fresh differentiation medium and cells were checked for transfection efficiency.

### CiliobrevinD washout assay

Cells were either treated with 50 μm CiliobrevinD or DMSO (control condition) at Day 1 or Day 4. 72 hours later (Day 4 or day 7), cells were treated as follows: Control cells in DMSO are washed and put in fresh differentiation medium with DMSO again and agrin, cells which were not washed out from ciliobrevinD were put in fresh differentiation medium with agrin and ciliobrevinD (ciliobrevinD condition) and washout cells were put in fresh differentiation with agrin and without ciliobrevinD (washout condition). 48 hours later (Day 6 or Day 9) cells were washed twice, fixed in PFA 4% and stained. The current conditions used for the ciliobrevinD have been chosen after testing several conditions (different concentrations, different time scale) which turned out to be either ineffective or to kill the cells massively.

### Nocodazole assay

Cells were either treated with 1 μg/ml nocodazole or with 0.25 μg/ml nocodazole and DMSO (control condition) at Day 4. Condition 1 μg/ml: Cells were incubated with 1 μg/ml nocodazole or DMSO for two hours. Nocodazole-treated and control cells were then washed briefly once in PBS1X, fixed and stained. Condition 0.25 μg/ml: Cells were incubated with 0.25 μg/ml nocodazole or DMSO. 72 hours later (Day 7), cells were treated as follows: nocodazole-treated and control were washed twice in PBS1X and put in fresh differentiation medium+agrin, and left to recover. 48 hours later (Day 9) cells were washed twice in PBS1X, fixed in PFA 4% and stained.

### Primary Antibodies

The following antibodies were used: mouse anti-DIC (Abcam clone 74.1, 1/100), rat ascites against tyrosinated α-tubulin was from the European Collection of Animal cell cultures (Salisbury, United Kingdom clone YL 1/2, 1/100), mouse anti-DHPR (Abcam ref Ab2864, 1/400), rabbit anti-phosphorylated MuSK (Origene ref TA314219 anti-Tyr755 ref, 1/100), anti-rabbit anti-MuSK (serum T194, gift obtained from Markus Ruegg, Biozentrum, University of Basel, 1/500), mouse anti-rapsyn (Abcam ref ab11423 (1234), 1/200), rabbit anti-rab4a Santa Cruz ref SC312, 1/100), rabbit anti-Rab7 (Santa Cruz, ref SC10767, 1/100), rabbit anti-Rab11 (Santa Cruz, ref SC9020, 1/100), mouse anti-myosin heavy chain (clone MF-20 DSHB Hybridoma Bank ref MF-20, 1/500), mouse anti-α-actinin (Sigma Aldrich ref A5044, 1/500), mouse anti-Giantin (Covance ref PRB-114C, 1/500), mouse anti-myogenin (DSHB Hybridoma Bank ref F5D, 1/200), mouse anti-GAPDH (clone 71.1, Sigma Aldrich, ref 8795, 1/1000).

### EDL muscle fiber isolation

EDL single fibers were isolated as described^71^. EDL muscle was explanted from symptomatic (Day 120) male or female SOD1G93A mice and corresponding littermates and then digested in DMEM (Gibco Ref 41966-029) containing 0.2% type I collagenase (Sigma ref C1030) for 2 h at 37 °C. Mechanical dissociation of fibers was performed using a thin pasteur pipette and followed under a transilluminating-fluorescent stereomicroscope.

### Fixation and Immunocytochemistry

The same protocol for cultured cells or isolated fibers was used. Cells were washed twice with PBS (Gibco ref 14190-094). Fixation was done in either 4% PFA (Electron Microscopy Science ref 15710-S) for 20 minutes at room temperature (RT) or in acetone/methanol solution (ratio 1:1) for 6 minutes at −20 °C accordingly to antibodies’ specific requirements. α-bungarotoxin labeling of AChRs was performed with 5 μg/ml TRITC-BTX (Sigma Aldrich ref T0195) in PBS for 15 minutes at RT prior to permeabilization and prior to acetone/methanol fixation. Following permeabilization in PBS 5%Triton (Sigma Aldrich ref X100) for 5 minutes at RT, cells were washed twice in PBS and then saturated in BSA 5%, 10% goat serum (Gibco ref 16210-064) for 1 hour at RT. Primary antibodies were incubated overnight at 4 °C in PBS BSA 5%, saponin 0.1%. Cells were washed with PBS at RT and stained with corresponding secondary antibodies supplemented with DAPI for 1 hour at RT. Cells were washed with PBS at RT and then mounted in Fluoromount medium (Fluromount-G, Southern Biotech, ref 0100-01) and analyzed.

### Quantitative real time PCR (qRT-PCR)

Total RNA was extracted from *in-vitro* differentiated fibers at Day 10 with TRIzol Reagent (Life Technologies). Genomic DNA removal and cDNA synthesis were performed using Quantitect Reverse Transcription Kit (Qiagen) with 300 ng of RNA as input. Gene expression was assessed with a LightCycler 96 Real-Time PCR System (Roche) using LightCycler 480 SYBR green I Master (Roche) with specific primers. Transcript levels were determined by absolute quantification using a 4-point standard curve and relative gene expression was calculated by normalization against cyclophilin reference genes. Sequences of the primers used for real-time PCR were as follows: DHC (Dync1h1 primer 1)-Fwd, 5′-AAGCACCTGCGTAAGCTGG3-′; DHC (Dync1h1)-Rev, 5′-GCGGGTCTGACAGGAACTTG-3′; (Dync1h1 primer 2)-Fwd, 5′-GGGATGAGTATGCCACGCTG-3′; DHC (Dync1h1)-Rev, 5′ TGTCCTTGAGCCCCTCTGAG-3′; MuSK-Fwd, 5′-CCCTGCAAGTGAAGATGAAA-3′; MuSK- Rev, 5′-TTCAAGAACTGCGATTCTGG-3′; AChRalpha- Fwd, 5′ GTAGAACACCCAGTGCTTCCA 3′; AChRalpha-Rev, 5′-GCCCGACCTGAGTAACTTCAT-3′; Rapsyn-Fwd, 5′-ATATCGGGCCATGAGCCAGTAC-3′; Rapsyn-Rev, 5′-TCACAACACTCCATG GCACTGC-3′; Lrp-4-Fwd, 5′-GCACACGGAATAGCCAGCA-3′; Lrp-4-Rev, GGATACAGGTACATTCGCCAAG Cyclophilin-Fwd, 5′-AAGAAGATCACCATTTCCGACT-3′; Cyclophilin-Rev, 5′-TTACAGGACATTGCGAGC-3′.

### Western blotting

*In vitro*-differentiated myofibers were lysed in PBS +1% SDS and passed through a Qiashredder column (Qiagen) to remove DNA. Protein concentration was measured with a BCA kit according to manufacturer instructions (Thermo Fischer Scientific). Equal amount of sample were boiled in 30 μl sample buffer and were loaded on 4–12% precast Bis‐Tris gel (Biorad) and transferred into nitrocellulose membrane using the TransBlot apparatus (Biorad). Membranes were blocked with blocking buffer (5% non‐fat dry milk, 0.1% Tween in TBS). Primary antibodies were incubated overnight in blocking buffer at 4 °C. After three washes with TBS‐Tween 0.1%, membranes were incubated with HRP‐conjugated secondary antibodies (1 h at room temperature). Proteins were visualized using ECL reagent (Pierce).

### Quantification of AChR cluster number and length

Myofibers were stained for AChR clusters with TRITC-α-bungarotoxin. Quantification method of AChR clusters was adapted from^33^. Clusters were defined as white patches of bright TRITC-α-bungarotoxin labeling with a length of equal to or greater than 10 μm. At least 120 myofibers in each condition were assessed and at least two independent experiments were performed. For SOD1^G93A^ myofibers, at least 60 fibers in each condition were assessed in at least two independent experiments. Error bars in plots represent standard deviation. P-values from student t-test.

### Quantification of peripheral nuclei, transversal triads and myofiber thickness

Quantification was performed as previously described^32^. Myofibers were stained for DHPR and DAPI and acquired with a Leica SPE confocal 40 × 1.15 NA ACS Apo at different Z positions. Nuclei extruding the myofiber periphery were considered as peripheral. Average of three measurements per fiber was calculated for myofiber thickness. A minimum of 40 fibers were counted per condition in at least two independent experiments. Error bars in plots represent standard deviation. P-values from student t-test.

### Quantification of Rapsyn, MuSK and phosphorylated MuSK at NMJ

Isolated fibers were stained for rapsyn, MuSK and phosphorylated MuSK. ROIs were defined at TRITC-BTX positive endplate. Binary images of TRITC-BTX positive endplate were created using Metamorph and the average intensity of rapsyn, MuSK and phosphorylated MuSK signal inside the endplate were quantified in Metamorph. A minimum of 4 isolated fibers were counted per condition in at least two different animals. Error bars in plots represent standard deviation. P-values from student t-test.

### Quantification of MuSK and Rab proteins colocalization

Myofibers were stained for MuSK and Rab 4/7/11 to mark the early, late and recycling endosomes respectively. ROIs were defined at MuSK cluster sites. Cytofluorogram of MuSK and Rab proteins and correlation coefficient of the MuSK and Rab protein signals at these ROIs were generated with the JaCoP plug-in of Image J. A minimum of 40 clusters were counted per condition in at least two independent experiments. Error bars in plots represent standard deviation. P-values from student t-test.

### Fluorescence

Epi-fluorescence images were acquired using a Nikon Timicroscope equipped with a CoolSNAP HQ2 camera (Roper Scientific), an XY-motorized stage (Nikon), driven by Metamorph (Molecular Devices) and 4 × 0.13NA, 10 × 0.30NA, 20 × 0.45NA, 40× PlanApo oil immersion objectives. Confocal images were acquired using Leica SPE confocal microscope with a 40 × 1.15 NA ACS Apo objective.

### Image analysis

Images were analyzed using Image J software or Metamorph 7.1 (Molecular Devices).

### Statistical analysis

Statistical significance was determined using GraphPad Prism (GraphPad Prism Software Inc. version 6). Statistical tests used are indicated in each method description. *represents P < 0.05; **represents P < 0.01 and ***represents P < 0.001. P < 0.05 was considered as significant.

## Additional Information

**How to cite this article**: Vilmont, V. *et al*. Dynein disruption perturbs post-synaptic components and contributes to impaired MuSK clustering at the NMJ: implication in ALS. *Sci. Rep*. **6**, 27804; doi: 10.1038/srep27804 (2016).

## Supplementary Material

Supplementary Information
