# Supplementary material for: Dynein disruption perturbs post-synaptic components and contributes to impaired MuSK clustering at the NMJ: implication in ALS
Source: Sci Rep. 2016 Jun 10;6:27804. doi: 10.1038/srep27804 (PMC4901269; doi:10.1038/srep27804)

Dynein disruption perturbs post-synaptic components and contributes to impaired MuSK clustering at the NMJ: implication in ALS

Valérie Vilmont[1], Bruno Cadot[1], Elsa Vezin[1], Fabien Le Grand[1] and Edgar R. Gomes[1,2]

[1] Myology Research Center, UM76-INSERM U974-CNRS FRE 361 Sorbonne Universités, UPMC Université Paris 06, Paris France

[2] Instituto de Medicina Molecular, Faculdade de Medicina, Universidade de Lisboa, Lisboa, Portugal

Online supplemental material

Supplementary Figures

Supplementary Figure 1. Characterization of myofibers in control vs *sh*DHC/ ciliobrevinD conditions.
A) Representative image of myofiber stained for α-BTX (green), Rapsyn (Red) and DAPI (blue).
B) Representative images of myofibers in control conditions vs *sh*DHC conditions stained for GFP (green), α-BTX (red), DIC (magenta) and DAPI (blue).
C) Western blot with dynein intermediate chain (DIC), p50 and GAPDH antibodies on primary in-vitro differentiated fibers, transfected with *sh*DHC or scramble myofibers. DIC intermediate chain is downregulated while dynactin p50 subunit is not affected.
D) Representative images of myofibers stained for Golgi (Giantin), *sh*RNA (GFP) and DAPI (blue). Fine dotted lines show cell periphery and coarse dotted lines represent outline of the Golgi apparatus. Golgi apparatus is dispersed in *sh*DHC transfected cells.
E) Quantification of Rapsyn clusters number per fiber and Rapsyn cluster length in control vs *sh*DHC conditions at 2 differentiation time points (days 6 and 9).
F) RT-qPCR analysis of AChR gene expression in in-vitro differentiated myofibers after *sh*RNA treatment. Error bars indicate SEM, n=4
G) RT-qPCR analysis of rapsyn gene expression in in-vitro differentiated myofibers after *sh*RNA treatment. Error bars indicate SEM, n=4
H) Timeline of ciliobrevinD washout assay.
I) Quantification of Rapsyn clusters number per fiber and Rapsyn cluster length in ciliobrevinD washout assay at 2 differentiation time points (days 6 and 9).
Scale bar 10µm.

Supplementary Figure 2. Effect of shDHC on AChR clustering genes and muscle differentiation.
A) Representative images of myofibers in ciliobrevinD washout assay stained for dynein intermediate chain (gray) and DAPI (blue).
B) Representative images of myofibers in control vs ciliobrevinD-treated conditions stained for α-tubulin (gray) and DAPI (blue).
C) Representative images of myofibers in control conditions vs *sh*DHC conditions at day 6 of differentiation stained for GFP (green), alpha-actinin (red) and DAPI (blue).

D) Representative images of myofibers in ciliobrevinD washout assay fixed at day 6 of differentiation stained for GFP (green), alpha-actinin (red) and DAPI (blue).
E) Representative images of myofibers in in control conditions vs *sh*DHC conditions at day 6 of differentiation stained for GFP (green), MF-20 (gray) and DAPI (blue).
F) Representative images of myofibers in ciliobrevinD washout assay fixed at day 6 of differentiation stained for MF-20 (gray) and DAPI (blue).

Supplementary Figure 3. Characterization of myofiber in ciliobrevinD-treated conditions and in presence of nocodazole.
A) RT-qPCR analysis of MuSK gene expression in in-vitro differentiated myofibers after *sh*RNA treatment. Error bars indicate SEM, n=4.
B) Representative image of myofiber stained for α-BTX (green), MuSK (Red) and DAPI (blue). Arrow represent AChR and MuSK cluster.
C) Representative image of myofiber with or without treatment with nocodazole at day 4 of differentiation (1µg/ml) stained for α-tubulin (gray) and DAPI (blue).
D) Representative images of myofibers with or without treatment with nocodazole day 4 of differentiation (1µg/ml) stained for acetylated-tubulin (gray) and DAPI (blue).
E) Representative images of myofibers with or without treatment with nocodazole (0.25µg/ml) day 7 of differentiation stained for α-tubulin (gray) and DAPI (blue).
F) Representative image of myofiber washed out from nocodazole (0.25µg/ml) day 9 of differentiation stained for BTX (green), MuSK (red), alpha-tubulin (gray) and DAPI (blue).
Scale bar 10µm.

Supplementary Figure 4. Comparison between SOD1^{G93A} vs WT myofibers
A) Representative images of WT and SOD1^{G93A} myofibers at day 6 of differentiation stained for α-BTX (green), MuSK (Red) and DAPI (blue).
B) Representative images of WT and SOD1^{G93A} myofibers at day 6 of differentiation stained for α-BTX (green), phosphoMuSK (Red) and DAPI (blue). Arrow represent AChR and phosphoMuSK cluster.
C) Representative images of WT and SOD1^{G93A} myofibers at day 6 of differentiation stained for α-BTX (green), rapsyn (Red) and DAPI (blue).
Scale bar 10µm.

Supplementary Figure1

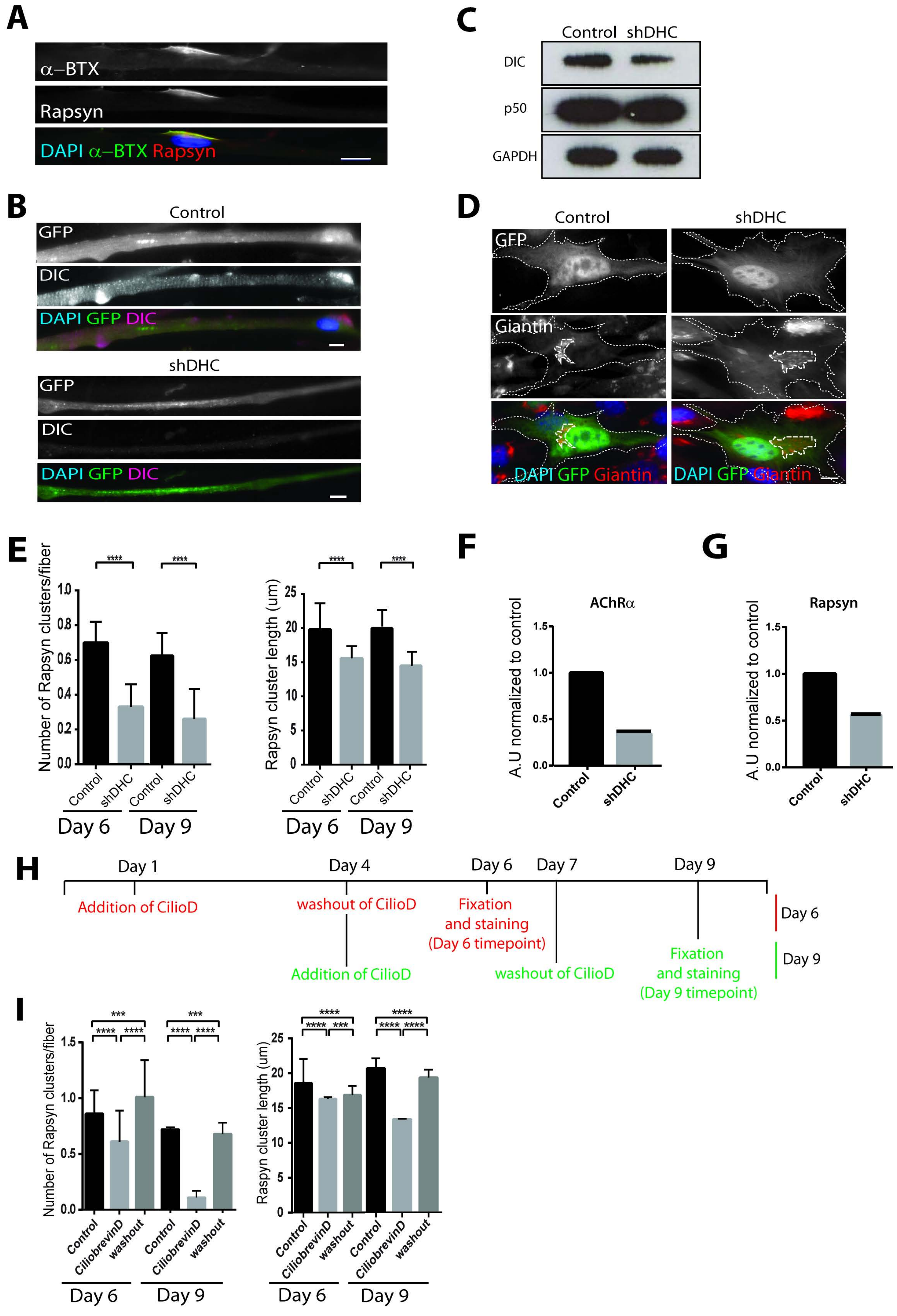

Supplementary Figure 2

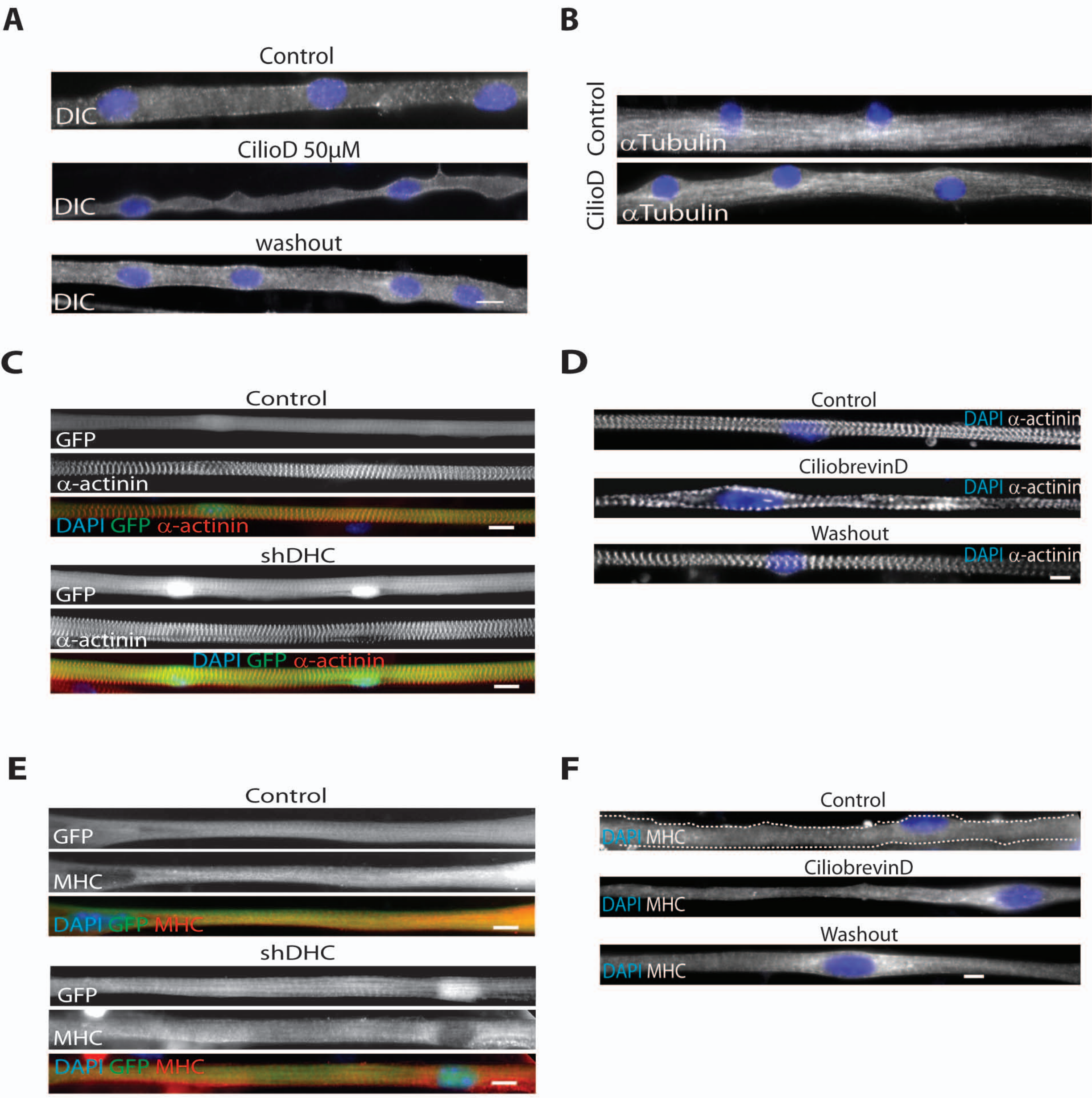

Supplementary Figure 3

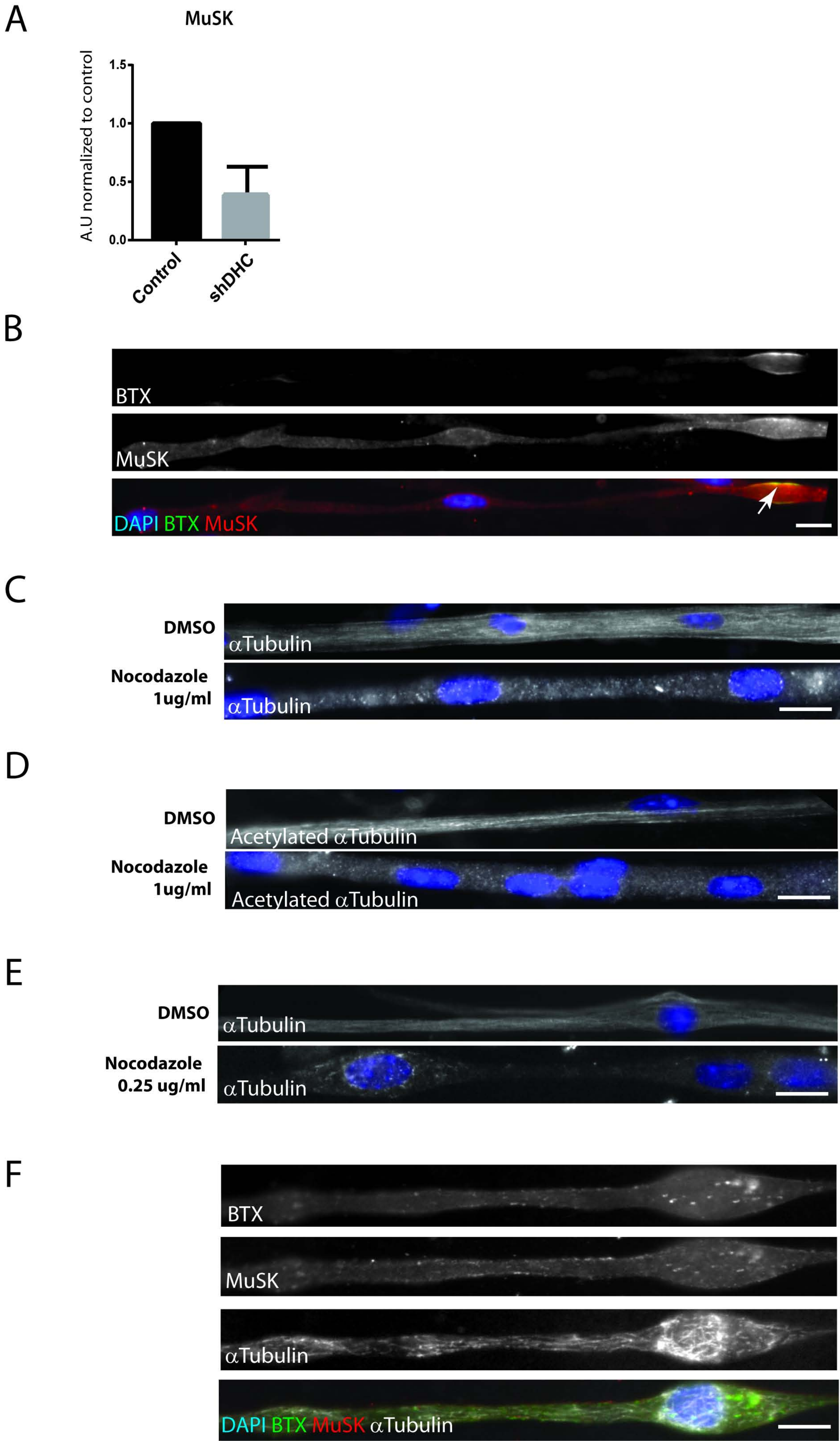

Supplementary Figure 4

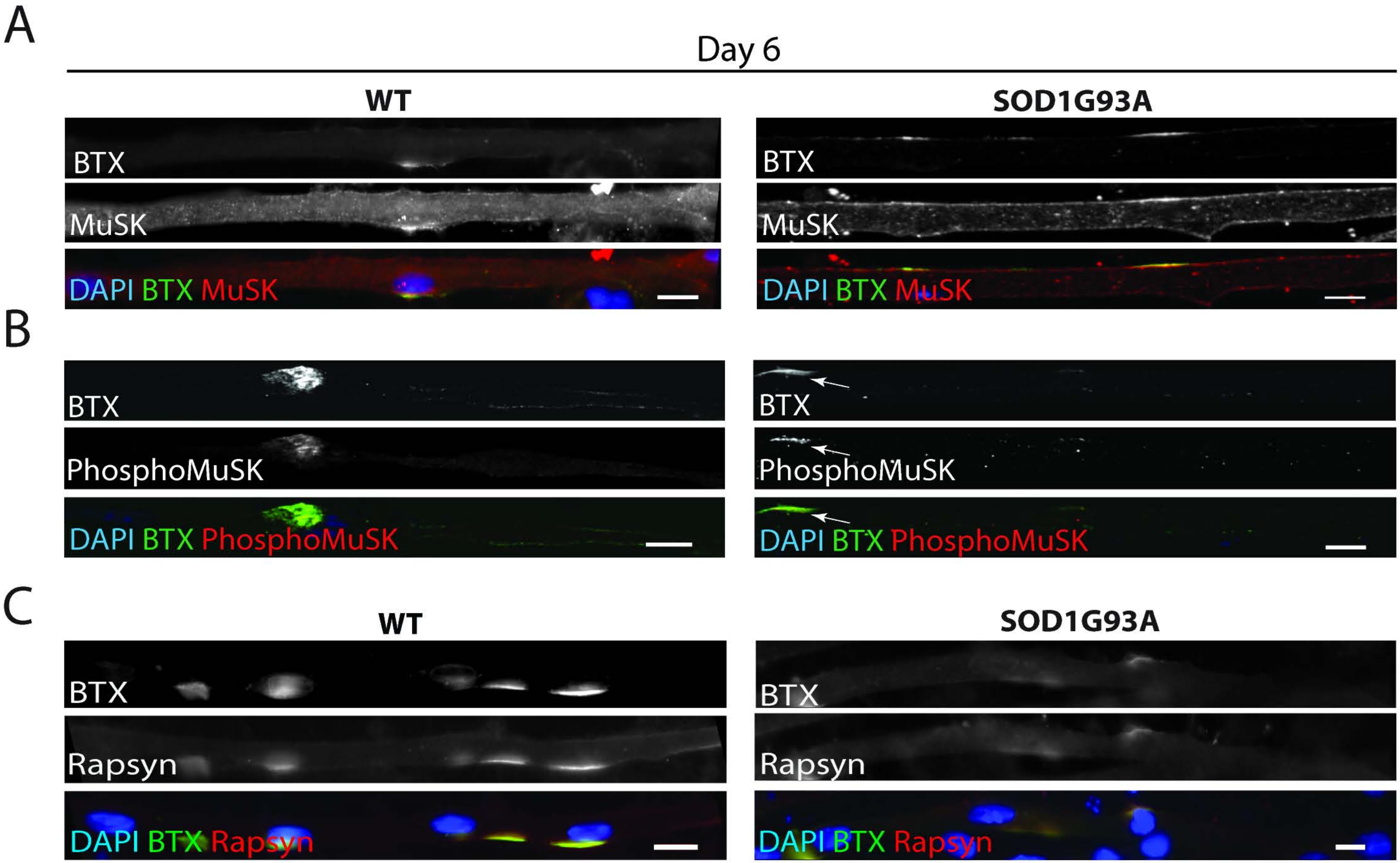

Supplement: Supplementary Information [file srep27804-s1.pdf]
